# Intramolecular Spodium Bonds in Zn(II) Complexes: Insights from Theory and Experiment

**DOI:** 10.3390/ijms21197091

**Published:** 2020-09-25

**Authors:** Mainak Karmakar, Antonio Frontera, Shouvik Chattopadhyay, Tiddo J. Mooibroek, Antonio Bauzá

**Affiliations:** 1 Department of Chemistry, Inorganic Section, Jadavpur University, Kolkata 700 032, India; mainak31893@gmail.com; 2 Departament de Química, Universitat de les Illes Balears, Crta. de Valldemossa km 7.5, 07122 Palma (Baleares), Spain; toni.frontera@uib.es; 3 van ‘t Hoff Institute for Molecular Sciences, Universiteit van Amsterdam, Science Park 904, 1098 XH Amsterdam, The Netherlands

**Keywords:** zinc complexes, spodium bonds, σ-hole interactions, CSD analysis, DFT calculations

## Abstract

Two new dinuclear zinc(II) complexes, [Zn_2_(*µ*_1,3_-OAc)(L^1^)_2_]I·MeOH (**1**) and [Zn_2_(*µ*_1,3_-OAc)(L^2^)(NCS)] (**2**), (where HL^1^ = 2-(((3-(dimethylamino)propyl)amino)methyl)-6-methoxy-phenol and H_2_L^2^ = 2,2′-[(1-Methyl-1,2-ethanediyl)bis(iminomethylene)]bis[6-ethoxyphenol]) have been synthesized and characterized by elemental and spectral analysis. Their X-ray solid state structures have been determined, revealing the existence of intramolecular Zn···O spodium bonds in both complexes due to the presence of methoxy (**1**) or ethoxy (**2**) substituents adjacent to the coordinated phenolic O-atom. These noncovalent interactions have been studied using density functional theory (DFT) calculations, the quantum theory of “atoms-in-molecules” and the noncovalent interaction plot. Moreover, a search in the Cambridge structure database (CSD) has been conducted in order to investigate the prevalence of intramolecular spodium bonds in Zn complexes. To our knowledge this is the first investigation dealing with intramolecular spodium bonds.

## 1. Introduction

Noncovalent interactions are very important in many fields of research, including molecular recognition, crystal engineering and catalysis [1,2,3]. Among the great deal of noncovalent forces, investigations on σ-hole interactions [4,5] are growing very fast and are definitively recognized by the scientific community as an alternative to the ubiquitous hydrogen bonding [6]. Very recently, the attractive interaction between elements of Group 12 of the Periodic Table and any electron rich ‘accepting’ atom (:A) [7,8] has been termed as a spodium bond (SpB) [9]. Therefore, the SpB has become a new member of the σ-hole family of interactions and it is adequate to differentiate the coordination bond (high covalent character) typical of transition metals from the noncovalent contact. SpBs are directional, the electron rich atom is located at distances that are longer than the sum of covalent radii and they are considerably weaker than coordination bonds. Moreover, the participation of the antibonding σ*(Sp–Y, where Y can be any atom) in the SpB bonding interaction (Y–Sp···:A) has been evidenced [9], as is common in σ-hole interactions [4,5].

This manuscript reports the synthesis and X-ray characterization of two new Zn(II) complexes that exhibit intramolecular SpBs. The utilization of two different tridentate and tetradendate ligands allows analyzing the interaction in two different Zn-coordination modes, square-pyramidal in (**1**) and pseudotetrahedral in (**2**). The SpBs in both compounds have been studied using density functional theory (DFT) calculations and characterized by a combination of the quantum theory of atoms in molecules (QTAIM) [10] and the noncovalent interaction (NCI) method [11]. Moreover, the Cambridge Structural Database (CSD) [12] has been examined in order to investigate the prevalence of intramolecular SpBs in Zn(II) complexes and their geometric features.

## 2. Results and Discussion

### 2.1. Synthesis

**Figure 1:**
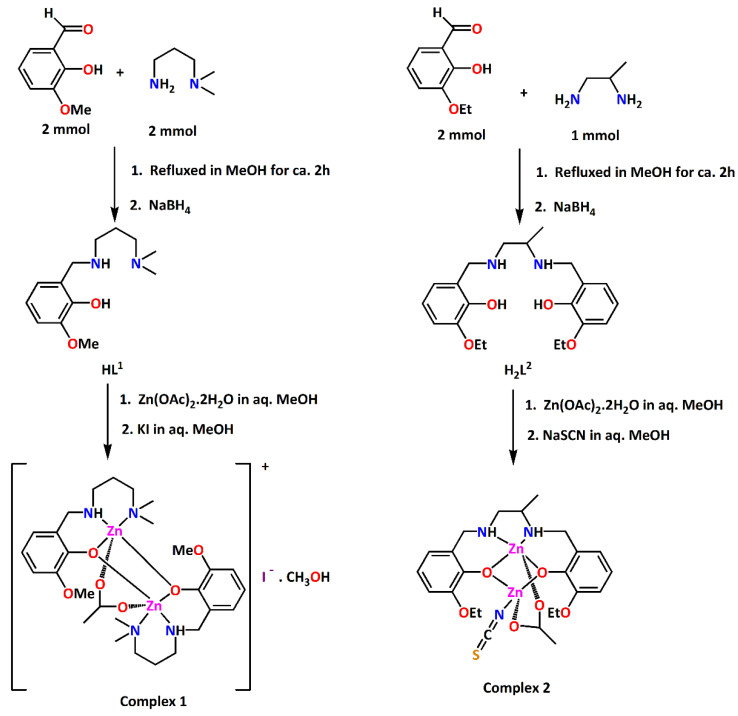
Synthetic routes to complexes **1** and **2**.

In this work, two acetate bridged dinuclear zinc complexes have been synthesized using reduced Schiff bases HL^1^ and H_2_L^2^ as ligands. HL^1^ was prepared by 1:1 condensation of 3-methoxysalicylaldehyde and *N*,*N*-dimethyl-1,3-diaminopropane in methanol and subsequent reduction using NaBH_4_ following a literature method [13,14,15]. In the same way, H_2_L^2^ was prepared by 2:1 condensation of 3-ethoxysalicylaldehyde and *rac*-1,2-diaminopropane in methanol followed by the reduction with NaBH_4_. Methanol solution of HL^1^ on reaction with zinc acetate dihydrate and potassium iodide in a 2:2:1 molar ratio produced complex **1**. On the other hand, methanol solution of H_2_L^2^ on reaction with zinc acetate dihydrate and sodium thiocyanate in a 1:2:1 molar ratio produced complex **2**. The synthetic routes to the complexes **1** and **2** are shown in Figure 1.

### 2.2. Description of Compounds ***1*** and ***2***

**Figure 2:**
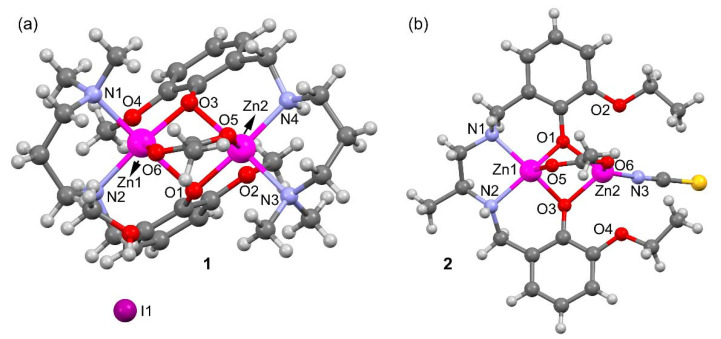
Perspective ball and stick view of the complexes **1** (**a**) and **2** (**b**) along with selective atom numbering scheme (only the coordinating atoms are labeled for clarity).

A perspective view of complexes **1** and **2** along with selective atom numbering scheme is depicted in Figure 2a,b respectively. **1** crystallizes in the orthorhombic space group *P*2_1_2_1_2_1_ and its asymmetric unit consists of an acetato bridged dinuclear zinc(II) L^1^_2_ complex, one I^–^ counter anion and a methanol solvent molecule. Each zinc is coordinated to the tridentate L^1^ via two amine N-atoms, one bridging acetate ligand and two bridging phenolate oxygen atoms (see Supplementary Figure S1). Both penta-coordinated zinc(II) centers are in a distorted square pyramidal geometry (see Supplementary Figures S2 and S3). The asymmetric unit contains one lattice methanol molecule that is H-bonded to the I^−^ counter anion. The counter ion is also H-bonded to N4–H4 of the reduced Schiff base ligand.

Compound **2** (Figure 2b and Supplementary Figure S4) crystallizes in the triclinic space group Pī and does not contain additional ions or solvent molecules in its asymmetric unit. Zn2 is N-coordinated to the thiocyanate, connected to O6 of the bridging acetate and bound to two bridging phenolate O-atoms to form a distorted tetrahedron (see Supplementary Figure S5). Zn1 is situated in a distorted square pyramidal environment where the ligand [O1,N1,N2,O3] donor atoms form the base of the pyramid and O5 of the bridging acetate is at the apex. The coordination distances of compounds **1** and **2** are given in Table 1 and range from 1.92 to 2.21 Å. These distances are similar or slightly longer that the sum of covalent radii (ΣR_cov_ = 1.93 and 1.86 Å for Zn+N and Zn+O, respectively) and can be considered as normal [16].

**Figure 3:**
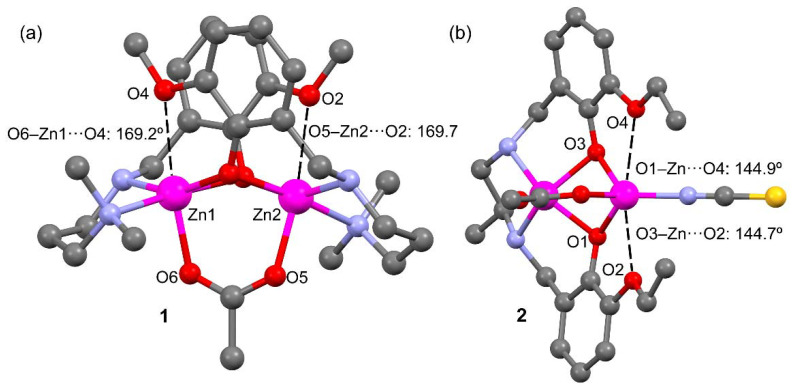
Spodium bonds (black dashed lines) and interacting angles in compounds **1** (**a**) and **2** (**b**). H-atoms omitted for clarity.

Table 1 also gathers longer Zn···O distances (values in italics) where the O-atom belongs to the methoxy (in L^1^) or ethoxy (in L^2^) substituent of the aromatic ring. These distances are around 0.8 Å longer than ΣR_cov_ and, consequently, can be considered as intramolecular spodium bonds, as further analyzed below. These interactions are depicted in Figure 3 as black dashed lines. In compound **1** the SpBs are located opposite to the Zn–O(acetate) coordination bonds at an angle of ~170°. In compound **2**, the ethoxyde O-atoms are located opposite the phenoxy O’s at an angle of ~145°. These differences in O–Zn···O angles likely originate from the different geometries involved; the square pyramidal geometry in **1** leaves ample space for an additional donor atom (SpB) while the accessible space at the tetrahedral Zn in **2** is more restricted.

The supporting information (Supplementary Figures S6–S10) contains a more detailed structural description complexes **1** and **2** (Section 1), the Hirshfeld surface analysis (Section 2) and additional spectral characterizations (Section 3; IR, UV-vis and XRPD spectra).

### 2.3. Theoretical DFT Study

**Figure 4:**
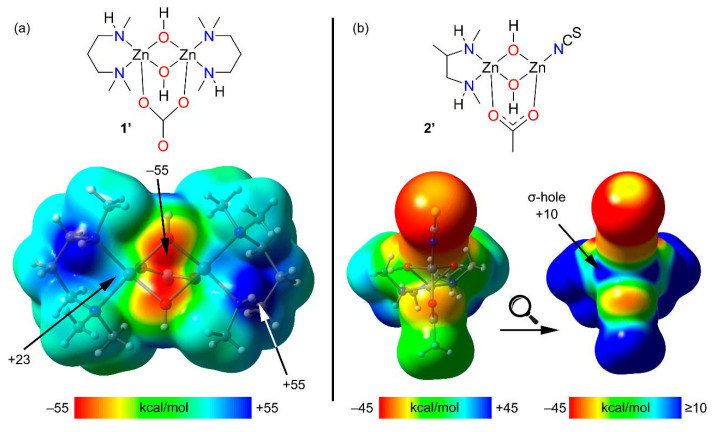
(**a**) Top: Model of compound **1** (named **1′**) used to investigate the existence of σ-holes. Bottom: MEP surface of **1′**; (**b**) Top: Model of compound **2** (named **2′**) used to investigate the existence of σ-holes. Bottom: MEP surfaces of **2′** (using two different MEP energetic scales). All isosurfaces have been computed at the PBE0-D3/def2-TZVP (isosurface 0.002 a.u.). The energies at selected points of the isosurfaces are given in kcal/mol.

Theoretical models of **1** and **2** (**1′** and **2′**, see Figure 4, top) have been used to investigate the existence of σ-holes at the Zn atoms. The utilization of such models is needed because in the real systems the potential σ-holes are “hidden” as a consequence of their intramolecular interaction with the electron rich O-atoms. In these models the aromatic rings were eliminated to leave hydroxides as bridging ligands instead of a phenoxides. The geometry of the models was optimized while keeping the zinc ions and their surrounding donor atoms frozen. For comparison purposes, the acetate bridging ligand in **1** has been replaced by a dianionic carbonate ligand in **1′** (i.e., so that both **1′** and **2′** are charge neutral).

The MEP surface of **1** shows that, as expected, the most positive MEP is located at the NH group. Additionally, two regions of positive potential are present at the extension of the O–Zn bonds (+23 kcal/mol), which is consistent with σ-holes adequate for interacting with electron rich atoms. The MEP value is most negative at the bridging O-atoms (−55 kcal/mol).

The MEP surface of **2′** on a default scale (bottom left in Figure 4b) is also most positive at the NH groups. Plotting the MEP on a different scale clearly reveals two patches of electropositive potential (+10 kcal/mol), congruent with the anticipated σ-holes.

**Figure 5:**
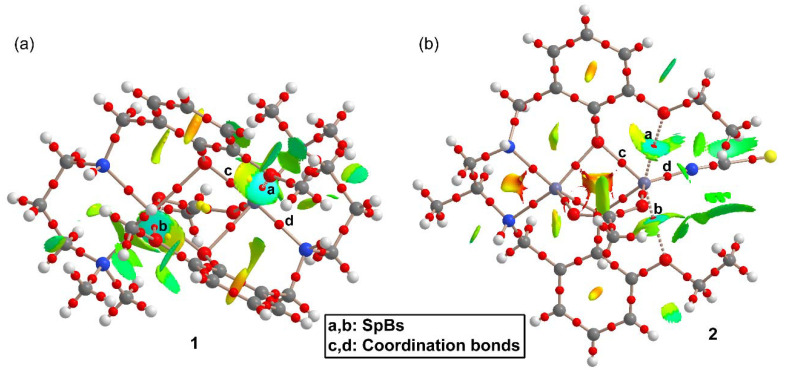
Quantum theory of atoms in molecules (QTAIM) and noncovalent interaction (NCI) method analyses combined in the same representation for compounds **1** (**a**) and **2** (**b**). Noncovalent bond paths are represented as dashed lines.

In order to characterize the SpBs in compounds **1** and **2**, we have performed combined QTAIM/NCI method analyses of both complexes, which are shown in Figure 5. Some surfaces, bond critical points (CPs) and bond paths in compound **1** corresponding to intramolecular C–H···π interactions have been omitted for clarity. Similarly, for compound **2** the critical points and bond paths of some intramolecular H-bonds involving the H-atoms of the ethoxy groups and the thiocyanate ligand have been omitted for clarity. The complete QTAIM/NCI method analyses of compounds **1** and **2** are shown in Supplementary Figure S11.

Figure 5a shows that each SpB in **1** is characterized by a bond CP and bond path connecting the O-atom to the Zn atom. Moreover, they are also characterized by blue isosurfaces in the NCI plot, thus evidencing an attractive interaction. The NCI isosurface extends toward the region in between the phenolic and methoxy O-atoms where the color changes to yellow, thus evidencing some repulsion between these atoms. It is also worth mentioning that similar isosurfaces are not present in the coordination bonds, due to their covalent character (covalent bonding is not revealed by the NCI method using the 0.004 cut-off for the electron density). In compound **2**, each SpB is characterized by bond CPs and bond path interconnecting the O-atom to the Zn-atom. The NCI analysis also shows two blue isosurfaces between the Zn and the O-atoms, evidencing attractive interactions. Similar to compound **1**, the isosurface extends toward the region between the O-atoms where the color is yellow, evidencing a repulsive O···O interaction. For both compounds, the bond CPs that characterize the SpBs have been labelled as “a” and “b” and two additional bond CPs that characterize Zn–O and Zn–N coordination bonds have been labelled as “c” and “d”. The QTAIM parameters measured at bond CPs a-d are summarized in Table 2. The values of ρ(r) and the Laplacian of ρ(r) at the bond CPs that characterize the SpBs are significantly smaller than those at the coordination bonds. In fact, the values at the bond CPs corresponding to SpBs are in the range of typical noncovalent interactions. For example, the densities of the bond CPs of about 0.02 is in between the 0.01 observed in a tetrel-bonding adduct and the 0.03 in a hydrogen-bonding adduct [17].

The same behavior is observed for the energy densities that are smaller for the SpBs compared to coordination bonds. Another interesting result is that the total energy density H(r), is negligible in the SpBs and negative in the coordination bonds, which is an indication of dominant covalent character in the coordination bonds. Finally, the delocalization index (DI) values, which are a measure of bond order, are also clearly different in coordination (ranging from 0.327 to 0.517) and spodium bonds (ranging from 0.059 to 0.075). Therefore, the QTAIM parameters is a convenient method to differentiate both types of bonding. 

**Figure 6:**
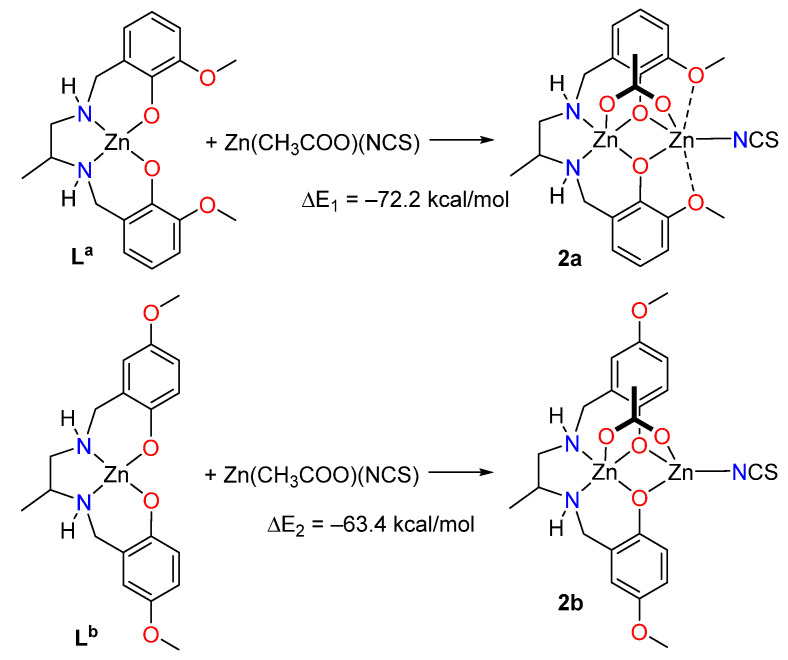
Reactions used to evaluate the SpB energy in compound **2**.

Two theoretical models have been used to evaluate energetically the SpBs in compound **2**. First, a slightly modified model of **2** (denoted as **2a**, see Figure 6) has been constructed where the ethoxy substituent has been changed by a methoxy group in order to eliminate the H-bonds between the ethoxy and the thiocyanate group that are present in **2** (see Supplementary Figure S11). Secondly, a hypothetical complex was calculated where the methoxy group is located *para* instead of *ortho* with respect to the phenolate O-atom (denoted as **2b**). In this complex the spodium bonds cannot be formed, while the basicity of the phenolate atoms is similar to that of **2a**. To estimate the intramolecular SpB energy, the formation energies of complexes **2a** and **2b** from the reaction of the metalloligands L^a^ and L^b^ (see Figure 1) with Zn(CH_3_COO)(NCS) have been computed. Secondly, the difference ΔE_1_ − ΔE_2_ can be attributed to the contribution of the SpBs. This difference is −8.8, −9.2 and −11.2 kcal/mol using, respectively, the PBE0-D3, B3LYP-D3 and MP2 methods. Therefore, each SpB stabilizes the complex with about −5 kcal/mol, which is in the range of binding energies recently estimated for intermolecular SpBs [7]. 

**Figure 7:**
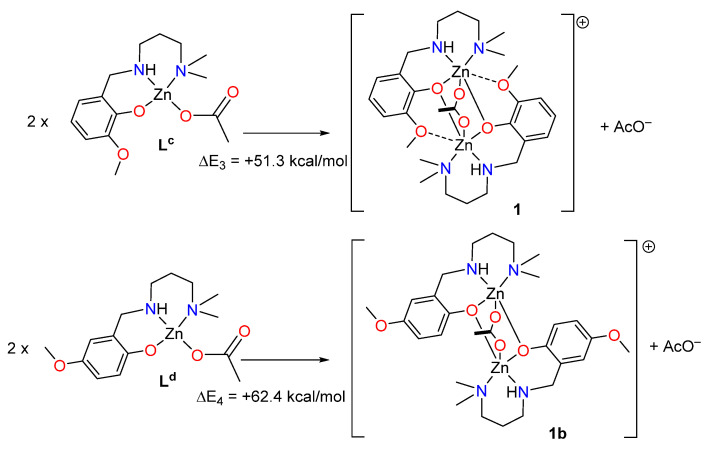
Reactions used to evaluate the spodium bond (SpB) energy in compound **1**.

Finally, two theoretical models have been also used to evaluate the SpBs in compound **1**. First, we have evaluated the formation of **1** from two molecules of the metalloligand denoted as L^C^ (see Figure 7). Secondly, a hypothetical complex was calculated where the methoxy group is located *para* instead of *ortho* with respect to the phenolate O-atom (denoted as **1b**). In this complex the spodium bonds cannot be formed, while the basicity of the phenolate atom is preserved. To estimate the intramolecular SpB energy, the formation energies of complexes **1** + AcO^−^ and **1b** + AcO^−^ from the reaction of two molecules of metalloligands L^c^ and L^d^ (see Figure 7) have been estimated, which are in this case endothermic. Secondly, the difference ΔE_1_ − ΔE_2_ can be attributed to the contribution of the SpBs, which is −11.1 kcal/mol. Therefore, each SpB stabilizes the complex in −5.55 kcal/mol, which is similar to the SpB bonding energy in **2**. 

### 2.4. CSD Search

**Figure 8:**
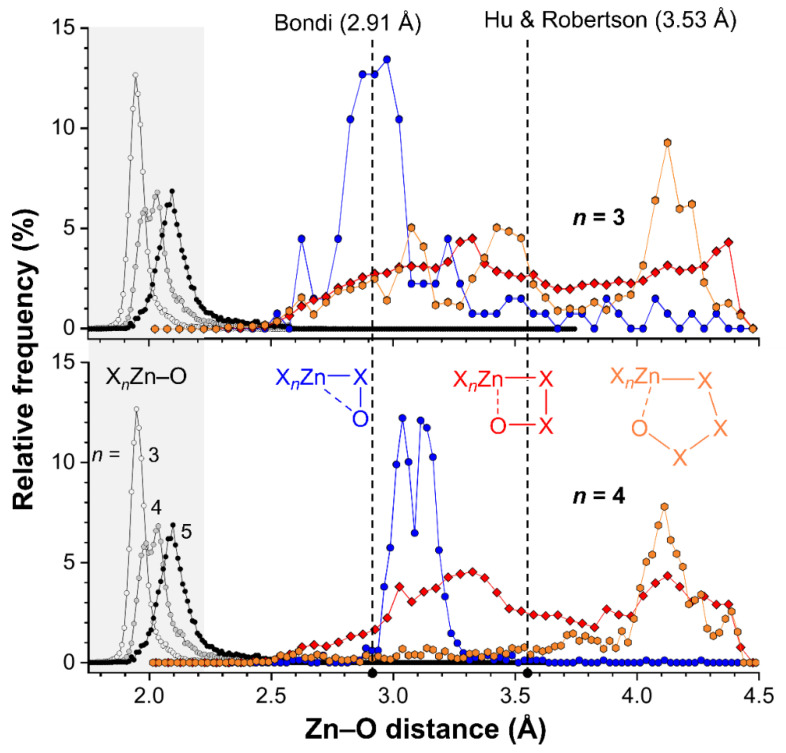
Plots of the relative frequencies as a function of the Intramolecular Zn···O distances for tetracoordinated (top) and pentacoordinated (bottom) Zn complexes where Zn and O are separated by two (blue circles), three (red diamonds) or four (orange hexagons) bonds. For reference purposes, the distribution of the Zn–O bond lengths found in complexes ZnX_4_ (white circles) ZnX_5_ (grey circles) or ZnX_6_ (black circles) are also plotted (highlighted in grey, X can be any atom). The dashed vertical lines indicate the sum of the van der Waals radii of O (1.52 Å) and Zn according to Bondi (1.39 Å) or Hu and Robertson (2.01 Å).

The CSD version 5.41 (including two updates until May 2020) was inspected using ConQuest version 2.0.4 (build 270014). All searches were limited to single crystal X-ray diffraction structures where 3D-coordinates were determined. In total, the CSD contains 22,055 Crystallographic Information Files (CIFs) with at least one SpX_4_ structure (entry 1a in Table 3), where Sp = Zn, Cd, Hg, X can be any atom, the Sp–X bonds were set at any type of bond and the number of bonded atoms to Sp were set to four (denoted by the “T4” superscript in the table). A similar search returned 22,449 CIFs for SpX_5_ structures (entry 2a) and 13,505 CIFs for SpX_6_ Structures (entry 3a). Within these three searches, the Zn–O bond distances were measured resulting in the data shown in entries 1b, 2b, and 3b for X_3_Sp–O, X_4_Sp–O, and X_5_Sp–O structures respectively. The resulting distance distributions have been plotted as relative frequencies as shown in Figure 8 (see grey highlighted area).

These distributions reveal clear peak-shapes that are centered around about 1.95 Å in tetracoordinated Zn (*n* = 3), 2.00 Å for pentacoordinated Zn (*n* = 4) and 2.10 Å for hexacoordinated Zn (*n* = 5). These distances are well below the sum of the van der Waals radii of O (1.52 Å) and Zn (1.39 Å according to Bondi [18,19] or 2.01 Å according to Hu and Robertson [20]; see also vertical dashed lines). The increased bond distances are likely a result of increased steric crowding of the Zn complexes with increasing coordination number. In all cases, virtually no data is found with a Zn–O bond distance above 2.5 Å.

To inspect the distance distributions of formally non-bonded Zn···O intramolecular distances, three distinct searched were performed for SpX_4_ (entry 1c–e in Table 3) and SpX_5_ structures (entry 2c–e in Table 3) where the Zn and O atoms were separate by two (entries c), three (entries d) or four (entries e) bonds. The cutoff for the intramolecular Zn···O distances was set at the sum of the van der Waals radii of Zn (1.39 Å) and O (1.52 Å) according to Bondi [18,19], plus a tolerance of 1.5 Å. The resulting distance distributions are also plotted in Figure 8 (top = ZnX_4_, bottom = ZnX_5_). Hardly any data was found with a Zn···O distance of 2.5 Å or below, implying that none of the cases found are genuine coordination bonds (found at ~2.0 Å, see grey highlight).

The data involving two bonds of separation between Zn and O are shown as blue spheres, and appear to display a grouping of data near 2.9 Å for ZnX_4_ and around 3.1 Å for ZnX_5_ structures. Both are well below the sum of the van der Waals radii according to Hu and Robertson (3.53 Å) and near the van der Waals benchmark according to Bondi (2.91 Å). It must be noted however, that an O atom removed two bonds away from Zn in a linear fashion (i.e., Zn–X–O = 180°) is found at about 2.9 Å, while this distance is 2.7 Å for a Zn–X–O angle of 90° (for X = N or O). This means that a relatively short distance (below the Hu and Robertson van der Waals benchmark) is inevitable, as is reflected by the grouping of data. The longer Zn···O distance observed for ZnX_5_ structures (bottom) is likely a result of the longer Zn–X distance on pentacoordinated structures (as found for Zn–O bonds).

The data for Zn···O distances separated by three bonds are shown as red diamonds and are nearly evenly distributed in between Zn···O = 2.5–4.5 Å with a small hill-like feature around 3.3 Å. Further inspection of these data revealed that most consist of carboxylates (OC(R)O; 5549/7880 CIFs for ZnX_4_ and 4778/6377 CIFs for ZnX_5_). Other ligands involve nitro groups, amides, perchlorates, phosphates and polyoxometallates. In these structures, a short Zn···O distance is not inevitable. For example, a simple molecular mechanics model of a η^1^-coordinated carboxylate ligand indicates than the uncoordinated Zn···O distance (which are three bonds apart) can vary from about 4.1 Å to 3.1 Å. The data below about 3.5 Å (the Hu and Robertson van der Waals benchmark) could thus be seen as cases of intramolecular interactions. Manual inspection of these data revealed that these features are an artifact arising largely from uncoordinated carboxylate O-atoms in five-membered chelate Zn-structures.

Data involving four bonds in between Zn and O are shown as orange hexagons and for both ZnX_4_ (top) and ZnX_5_ (bottom) structures there is a feature at about 4.1 Å. This feature cannot be ascribed to an interaction as this is above the van der Waals benchmarks (according to both Bondi and Hu and Robertson). For the data involving tetracoordinated Zn (top) there is some data in between 2.5–3.7 Å that is consistent with an interaction geometry. Data with such short distances are less prevalent for structures involving pentacoordinated Zn (bottom).

Given the analyses above it is clear that there is no strong directional trend in the intramolecular SpB for Zn···O interactions. Moreover, the Zn···O distances found in **1** (2.664 and 2.692 Å) and **2** (2.667 and 2.688 Å) are on the short side of the distributions shown in Figure 8 and can thus be considered as rare. This is particularly relevant for the distributions with four bonds of separation between Zn and O (orange spheres), which is also the case in **1** and **2**. It must be noted however, that the 1,2-relationship of the two O-donor atoms in the ligands deployed preorganizes the alkoxy groups involved in the SpB interaction.

## 3. Materials and Methods

### 3.1. Materials

All chemicals were of reagent grade and used as purchased from Sigma-Aldrich without further purification.

### 3.2. Preparation of Reduced Schiff Base Ligands

#### 3.2.1. Preparation of 2-(((3-(dimethylamino)propyl)amino)methyl)-6-methoxyphenol (HL^1^)

For the synthesis of *HL^1^* a solution of *N*,*N*-dimethyl-1,3-diaminopropane (2 mmol, 0.25 mL) with 3-methoxysalicylaldehyde (2 mmol, 0.310 g) in methanol (20 mL) was refluxed for ca. 2 h. The resulting yellow colored solution (20 mL) was then cooled to 0 °C in an ice bath, and solid sodium borohydride (4 mmol, 0.150 g) was added slowly with constant stirring until the yellow color of the solution disappeared. The resulting reaction mixture was acidified with glacial acetic acid (3 mL) and then evaporated to dryness under reduced pressure. The resulting slurry was suspended in water (15 mL) and extracted with dichloromethane. Finally, the solvent (i.e., dichloromethane) was evaporated under reduced pressure in a rotary evaporator to obtain the reduced Schiff base ligand, HL^1^.

#### 3.2.2. Preparation of 2,2′-[(1-Methyl-1,2-ethanediyl)bis(iminomethylene)]bis[6-ethoxyphenol] (H_2_L^2^)

A methanol solution containing 1,2-diaminopropane (2 mmol, 0.17 mL) and 3-ethoxysalicylaldehyde (2 mmol, 0.330 g) was refluxed for ca. 2 h resulting in a yellow solution. NaBH_4_ (4 mmol, 0.150 g) was added and the reaction mixture was stirred the solution was colorless. The resulting reaction mixture was acidified with glacial acetic acid (3 mL) and the volatiles were evaporation under reduced pressure. The remaining mass was suspended in water (15 mL) and extracted with dichloromethane. Finally, the solvent (i.e., dichloromethane) was evaporated under reduced pressure in a rotary evaporator to obtain H_2_L^2^.

#### 3.2.3. Preparation of the Complex [Zn2(µ1,3-OAc)(L1)2]I·MeOH (**1**)

A methanol solution of zinc acetate dihydrate (2 mmol, 0.440 g) was added to a stirred methanol solution of the ligand HL^1^. Stirring was continued for an additional 2 h. An aqueous methanol (1:1) solution of potassium iodide (1 mmol, 0.170 g) was then added and the mixture was stirred for ca. 2 h. The solution was then kept in open air at room temperature for a few days, resulting in the formation of white crystals that were isolated by filtration. X-ray quality single crystals were collected from this crystalline product.

Yield: 0.550 g, 68% (based on zinc). Anal. Calc. for C_29_H_49_N_4_O_7_Zn_2_I (823.40): C, 42.30; H, 6.00; N, 6.80%. Found: C, 42.1; H, 5.9; N, 6.9%. FT-IR (KBr, cm^−1^): 3158 (ν_N−H_); 2997-2878(ν_C−H_); 1473 {ν_sym_ (COO^−^)}; 1557 {ν_asym_ (COO^−^)}. λ_max_ (nm) [ε_max_(lit mol^−1^ cm^−1^)] (DMF): 291 (6.25 × 10^3^).

#### 3.2.4. Preparation of the Complex [Zn2(µ1,3-OAc)(L2)(NCS)] (**2**)

A methanol solution of zinc acetate dihydrate (2 mmol, 0.440 g) was added to a stirred solution of ligand H_2_L^2^ in methanol. Stirring was continued for an additional 2 h. An aqueous methanol (1:1) solution of sodium thiocyanate (1 mmol, 0.080 g) was added and stirring was continued ca. 2 h. The resulting solution was kept in open air at room temperature for few days, resulting in the formation of a white crystalline product that could be isolated by filtration. X-ray quality single crystals were collected from this crystalline product.

Yield: 0.430 g, 69% (based on zinc). Anal. Calc. for C_24_H_31_N_3_O_6_SZn_2_ (620.35): C, 46.47; H, 5.04; N, 6.77%. Found: C, 46.3; H, 4.9; N, 6.9%. FT-IR (KBr, cm^−1^): 3223 (ν_N−H_); 2979-2878 (ν_C−H_); 2102 (ν_NCS_); 1472 {ν_sym_ (COO^−^)}; 1566 {ν_asym_ (COO^−^)}. λ_max_ (nm) [ε_max_(lit mol^−1^ cm^−1^)] (DMF): 286 (6.93 × 10^3^).

### 3.3. Details of Instrumentation

Elemental analysis (carbon, hydrogen and nitrogen) was performed using a Perkin-Elmer 240C elemental analyzer. IR spectrum in KBr (4500–500 cm^−1^) was recorded with a Perkin-Elmer Spectrum Two spectrophotometer. Electronic spectra in DMF were recorded on a JASCO V-630 spectrophotometer. The powder XRD data were collected on a Bruker D8 Advance X-ray diffractometer using Cu K_α_ radiation (λ = 1.548 Å) generated at 40 kV and 40 mA. The PXRD spectrum was recorded in a 2θ range of 5–50° using 1-D Lynxeye detector at ambient conditions.

### 3.4. Crystal Data Collection and Refinement

Suitable single crystals of both complexes were used for data collection as described earlier [21]. Direct methods were employed for determination of molecular structures and refinements were done by full-matrix least square methods using the SHELX-18 package [22]. Multi-scan empirical absorption corrections were accomplished using the SADABS program [23]. The details of crystallographic data and structure refinement parameters are provided in Table 4.

### 3.5. Theoretical Methods

The energies and geometries of the complexes included in this study were computed at the PBE0 [24]-D3 [25]/def2-TZVP [26] level of theory by means of the TURBOMOLE 7.0 software [27]. The Bader’s “Atoms in molecules” theory and NCI method [28] have been used to study the interactions discussed herein by means of the AIMall calculation package [29]. Calculations related to the wavefunction properties were carried out using the Gaussian 16 calculation package [30] at the same level of theory. In particular, the density and potential energy cubes used to generate the MEP surfaces and the wavefunction used as input to perform the QTAIM/NCI analyses were obtained using Gaussian-16. The PBE0-D3 level of theory has been recently used by us to analyze σ/π-hole interactions in the solid state [31,32,33,34,35,36,37,38]

## 4. Concluding Remarks

Two new dinuclear Zn(II) complexes have been synthesized and characterized. They exhibit intramolecular spodium bonds that have been described herein for the first time. Moreover, the intramolecular SpBs have been characterized and differentiated from coordination bonds using a combination of QTAIM and NCI method analyses. The existence of σ-hole in both tetracoordinated and pentacoordinated Zn(II) atoms has been evidenced using MEP surfaces. Due to the intramolecular nature of the interaction, the strength of the spodium bond in **2** has been estimated using two theoretical models that evidence a moderately strong interaction (about −5 kcal/mol). Finally, the CSD analysis of intramolecular spodium bonds revealed that the short Zn···O interactions observed in compounds **1** and **2** are rare and likely largely affected by the pre-organization of the ligand.

## Figures and Tables

**Table 1:** Selected bond lengths (Å) in compounds **1** and **2**.

**1**			
Zn1–N1 (coord.) ^1^	2.169(5)	Zn2–N3 (coord.)	2.160(5)
Zn1–N2 (coord.)	2.074(4)	Zn2–N4 (coord.)	2.085(4)
Zn1–O1 (coord.) ^2^	2.212(4)	Zn2–O1 (coord.)	1.983(4)
Zn1–O3 (coord.)	1.977(3)	Zn2–O3 (coord.)	2.165(3)
Zn1–O6 (coord.)	2.051(4)	Zn2–O5 (coord.)	2.075(4)
*Zn1–O4 (SpB)*	*2.688(5)*	*Zn2–O2 (SpB)*	*2.667(4)*
Zn1···Zn2	3.047(1)		
**2**			
Zn1–N1 (coord.)	2.091(2)	Zn2–N3 (coord.)a	1.922(2)
Zn1–N2 (coord.)	2.099(2)	Zn2–O1 (coord.)	2.017(1)
Zn1–O1 (coord.)	2.058(2)	Zn2–O3 (coord.)	2.007(2)
Zn1–O3 (coord.)	2.042(2)	Zn2–O6 (coord.)	1.976(2)
Zn1–O5 (coord.)	1.978(2)	*Zn2–O2 (SpB)*	*2.692(2)*
Zn1···Zn2	2.9025(5)	*Zn2–O4 (SpB)*	*2.664(2)*

^1^ Sum of Zn and N covalent radius: 1.93 Å; ^2^ Sum of Zn and O covalent radius: 1.86 Å.

**Table 2:** Electron charge density (ρ), its Laplacian (∇^2^ρ), kinetic (V), Lagrangian (G) and total (H) energy densities at the bond critical points (CPs) labelled in Figure 5 for compounds **1** and **2** in a.u.

CP	ρ(r)	∇^2^ρ(r)	V(r)	G(r)	H(r)	DI
**1**						
**a**	0.017	0.055	−0.014	0.014	0.000	0.072
**b**	0.018	0.057	−0.015	0.015	0.000	0.075
**c**	0.081	0.428	−0.136	0.121	−0.015	0.370
**d**	0.076	0.313	−0.107	0.093	−0.014	0.394
**2**						
**a**	0.016	0.055	−0.014	0.014	0.000	0.059
**b**	0.017	0.059	−0.015	0.015	0.000	0.064
**c**	0.073	0.388	−0.117	0.107	−0.010	0.327
**d**	0.099	0.475	−0.171	0.145	−0.026	0.510

**Table 3:** Numerical overview of CSD data. Sp = Zn, Cd or Hg, X = any atom, all bonds could be any type of bond.

Entry	Search	CIFs	Hits
**1a**	Sp^T4^X_4_	22,055	
**1b**	⮡ X_3_Zn^T4^–O	9557	37,255
**1c** ^a^	⮡ X_3_Zn^T4^–X–O (2 bonds)	62	134
**1d** ^ a,b^	⮡ X_3_Zn^T4^–X–X–O (3 bonds)	7880	31,120
**1e** ^a^	⮡ X_3_Zn^T4^–X–X–X–O (4 bonds)	724	2123
**2a**	Sp^T5^X_5_	22,449	
**2b**	⮡ X_4_Zn^T5^–O	8649	36,519
**2c** ^a^	⮡ X_4_Zn^T5^–X–O (2 bonds)	240	818
**2d** ^a,c^	⮡ X_4_Zn^T5^–X–X–O (3 bonds)	6377	21,227
**2e** ^a^	⮡ X_4_Zn^T5^–X–X–X–O (4 bonds)	1136	2463
**3a**	Sp^T6^X_6_	13,505	
**3b**	⮡ X_5_Zn^T6^–O	10,347	44,770

^a^ The intramolecular Zn···O distance was limited to 4.41 Å (i.e., The sum of the Bondi van der Waals radii of Zn (1.39 Å) and O (1.52 Å) plus a tolerance of 1.5 Å); ^b^ 5549 Crystallographic Information Files (CIFs) involve a carboxylate (OC(R)O) fragment; ^c^ 4778 CIFs involve a carboxylate (OC(R)O) fragment.

**Table 4:** Crystal data and refinement details of complexes **1** and **2**.

Complex	1	2
Formula	C_29_H_49_N_4_O_7_Zn_2_I	C_24_H_31_N_3_O_6_SZn_2_
Formula Weight	823.40	620.37
Temperature (K)	273(2)	273(2)
Crystal System	Orthorhombic	Triclinic
Space group	*P*2_1_2_1_2_1_	*P* $\bar{1}$
*a* (Å)	12.744(6)	10.5900(8)
*b* (Å)	16.260(7)	11.7781(9)
*c* (Å)	17.008(8)	12.0470(9)
β (°)	90	90.588(2)
β (°)	90	101.556(2)
γ (°)	90	109.376(2)
Z	4	2
*d_cal_* (g cm^−3^)	1.552	1.489
μ(mm^−1^)	2.284	1.850
F(000)	1680.0	640
Total reflection	29800	49447
Unique Reflections	7136	6157
Observe data[I>2σ(I)]	6500	5165
R(int)	0.052	0.032
R1, *w*R2 (all data)	0.0392, 0.0675	0.0408, 0.1052
R1, *w*R2 [I>2*σ*(I)]	0.0338, 0.0655	0.0308, 0.0922

## Supplementary Materials

The supplementary materials are available online at https://www.mdpi.com/1422-0067/21/19/7091/s1.
