# Supplementary material for: Intramolecular Spodium Bonds in Zn(II) Complexes: Insights from Theory and Experiment
Source: Int J Mol Sci. 2020 Sep 25;21(19):7091. doi: 10.3390/ijms21197091 (PMC7582961; doi:10.3390/ijms21197091)

checkCIF/PLATON report

You have not supplied any structure factors. As a result the full set of tests cannot be run.

THIS REPORT IS FOR GUIDANCE ONLY. IF USED AS PART OF A REVIEW PROCEDURE FOR PUBLICATION, IT SHOULD NOT REPLACE THE EXPERTISE OF AN EXPERIENCED CRYSTALLOGRAPHIC REFEREE.

No syntax errors found. CIF dictionary Interpreting this report

Datablock: 1

```
Bond precision:  C-C = 0.0084 A          Wavelength=0.71073

Cell:              a=12.744(6)      b=16.260(7)      c=17.008(8)
                   alpha=90         beta=90          gamma=90
Temperature:       273 K
```

	Calculated	Reported
Volume	3524(3)	3524(3)
Space group	P 21 21 21	P 21 21 21
Hall group	P 2ac 2ab	P 2ac 2ab
Moiety formula	C28 H45 N4 O6 Zn2, C H4 O, I	?
Sum formula	C29 H49 I N4 O7 Zn2	C29 H49 I N3 O7 Zn2
Mr	823.40	823.40
Dx,g cm-3	1.552	1.552
Z	4	4
Mu (mm-1)	2.284	2.283
F000	1680.0	1680.0
F000’	1680.94	
h,k,lmax	15,20,21	15,20,21
Nref	7136[3980]	7136
Tmin,Tmax	0.264,0.504	0.264,0.504
Tmin’	0.155	

```
Correction method= # Reported T Limits: Tmin=0.264 Tmax=0.504
AbsCorr = MULTI-SCAN

Data completeness= 1.79/1.00      Theta(max)= 26.272

R(reflections)= 0.0338( 6500)     wR2(reflections)= 0.0675( 7127)

S = 1.087                 Npar= 399
```

The following ALERTS were generated. Each ALERT has the format
test-name_ALERT_alert-type_alert-level.
Click on the hyperlinks for more details of the test.

Alert level C

```
CHEMW01_ALERT_1_C  The ratio of given/expected molecular weight as calculated
            from the _chemical_formula_sum lies outside
            the range 0.99 <> 1.01
            Calculated formula weight =   809.3865
            Formula weight given      =   823.4000
CHEMW01_ALERT_1_C  The difference between the given and expected weight for
            compound is greater 1 mass unit. Check that all hydrogen
            atoms have been taken into account.
SHFSU01_ALERT_2_C  The absolute value of parameter shift to su ratio > 0.05
            Absolute value of the parameter shift to su ratio given   0.053
            Additional refinement cycles may be required.
PLAT041_ALERT_1_C Calc. and Reported SumFormula    Strings  Differ     Please Check
PLAT222_ALERT_3_C NonSolvent Resd 1  H   Uiso(max)/Uiso(min) Range        4.3 Ratio
PLAT245_ALERT_2_C U(iso) H2        Smaller than U(eq) N2        by      0.012 Ang**2
PLAT260_ALERT_2_C Large Average Ueq of Residue Including      O100      0.158 Check
PLAT342_ALERT_3_C Low Bond Precision on  C-C Bonds ...............    0.00837 Ang.
PLAT420_ALERT_2_C D-H Without Acceptor       N2       --H2       .     Please Check
PLAT420_ALERT_2_C D-H Without Acceptor       N4       --H4       .     Please Check
```

Alert level G

```
FORMU01_ALERT_2_G  There is a discrepancy between the atom counts in the
            _chemical_formula_sum and the formula from the _atom_site* data.
            Atom count from _chemical_formula_sum:C29 H49 I1 N3 O7 Zn2
            Atom count from the _atom_site data:  C29 H49 I1 N4 O7 Zn2
CELLZ01_ALERT_1_G Difference between formula and atom_site contents detected.
CELLZ01_ALERT_1_G ALERT: Large difference may be due to a
            symmetry error - see SYMMG tests
           From the CIF: _cell_formula_units_Z    4
           From the CIF: _chemical_formula_sum  C29 H49 I N3 O7 Zn2
           TEST: Compare cell contents of formula and atom_site data

           atom    Z*formula  cif sites diff
           C        116.00    116.00    0.00
           H        196.00    196.00    0.00
           I          4.00      4.00    0.00
           N         12.00     16.00   -4.00
           O         28.00     28.00    0.00
           Zn         8.00      8.00    0.00
PLAT002_ALERT_2_G Number of Distance or Angle Restraints on AtSite          4 Note
PLAT007_ALERT_5_G Number of Unrefined Donor-H Atoms ..............          1 Report
PLAT063_ALERT_4_G Crystal Size Possibly too Large for Beam Size ..       0.80 mm
PLAT066_ALERT_1_G Predicted and Reported Tmin&Tmax Range Identical          ? Check
PLAT172_ALERT_4_G The CIF-Embedded .res File Contains DFIX Records          2 Report
PLAT199_ALERT_1_G Reported _cell_measurement_temperature ..... (K)        273 Check
PLAT200_ALERT_1_G Reported   _diffrn_ambient_temperature ..... (K)        273 Check
PLAT380_ALERT_4_G Incorrectly? Oriented X(sp2)-Methyl Moiety .....        C28 Check
PLAT791_ALERT_4_G Model has Chirality at N2          (Sohnke SpGr)          R Verify
PLAT791_ALERT_4_G Model has Chirality at N4          (Sohnke SpGr)          R Verify
PLAT794_ALERT_5_G Tentative Bond Valency for Zn1       (II)      .       1.80 Info
PLAT794_ALERT_5_G Tentative Bond Valency for Zn2       (II)      .       1.80 Info
PLAT860_ALERT_3_G Number of Least-Squares Restraints ............          2 Note
PLAT883_ALERT_1_G No Info/Value for _atom_sites_solution_primary .     Please Do !
```

```
 0 ALERT level A = Most likely a serious problem - resolve or explain
 0 ALERT level B = A potentially serious problem, consider carefully
10 ALERT level C = Check. Ensure it is not caused by an omission or oversight
17 ALERT level G = General information/check it is not something unexpected

 9 ALERT type 1 CIF construction/syntax error, inconsistent or missing data
 7 ALERT type 2 Indicator that the structure model may be wrong or deficient
 3 ALERT type 3 Indicator that the structure quality may be low
 5 ALERT type 4 Improvement, methodology, query or suggestion
 3 ALERT type 5 Informative message, check
```

Datablock: 2

```
Bond precision:  C-C = 0.0048 A          Wavelength=0.71073

Cell:           a=10.5900(8)      b=11.7781(9)      c=12.0470(9)
                alpha=90.588(2)   beta=101.556(2)   gamma=109.376(2)
Temperature:    273 K

                Calculated                Reported
Volume          1383.99(18)               1383.99(18)
Space group     P -1                      P -1
Hall group      -P 1                      -P 1
Moiety formula  C24 H31 N3 O6 S Zn2       ?
Sum formula     C24 H31 N3 O6 S Zn2       C24 H31 N3 O6 S Zn2
Mr              620.36                    620.32
Dx,g cm-3       1.489                     1.489
Z               2                         2
Mu (mm-1)       1.850                     1.850
F000            640.0                     640.0
F000'           641.60
h,k,lmax        13,15,15                  13,15,15
Nref            6157                      6157
Tmin,Tmax       0.275,0.477               0.275,0.477
Tmin'           0.219

Correction method= # Reported T Limits: Tmin=0.275 Tmax=0.477
AbsCorr = MULTI-SCAN

Data completeness= 1.000          Theta(max)= 27.142

R(reflections)= 0.0308( 5165)     wR2(reflections)= 0.1052( 6140)

S = 1.181                  Npar= 333
```

The following ALERTS were generated. Each ALERT has the format
test-name_ALERT_alert-type_alert-level.
Click on the hyperlinks for more details of the test.

Alert level C

```
PLAT242_ALERT_2_C Low    'MainMol' Ueq as Compared to Neighbors of        C22 Check
PLAT242_ALERT_2_C Low    'MainMol' Ueq as Compared to Neighbors of        C24 Check
PLAT420_ALERT_2_C D-H Without Acceptor       N2       --H1N      .     Please Check
```

Alert level G

```
PLAT063_ALERT_4_G Crystal Size Possibly too Large for Beam Size ..       0.80 mm
PLAT066_ALERT_1_G Predicted and Reported Tmin&Tmax Range Identical          ? Check
PLAT154_ALERT_1_G The s.u.'s on the Cell Angles are Equal ..(Note)      0.002 Degree
PLAT199_ALERT_1_G Reported _cell_measurement_temperature ..... (K)        273 Check
PLAT200_ALERT_1_G Reported   _diffrn_ambient_temperature ..... (K)        273 Check
PLAT380_ALERT_4_G Incorrectly? Oriented X(sp2)-Methyl Moiety .....        C23 Check
PLAT793_ALERT_4_G Model has Chirality at N1         (Centro SPGR)          S Verify
PLAT793_ALERT_4_G Model has Chirality at N2         (Centro SPGR)          R Verify
PLAT793_ALERT_4_G Model has Chirality at C11        (Centro SPGR)          S Verify
PLAT794_ALERT_5_G Tentative Bond Valency for Zn1       (II)      .       1.99 Info
PLAT794_ALERT_5_G Tentative Bond Valency for Zn2       (II)      .       1.93 Info
PLAT883_ALERT_1_G No Info/Value for _atom_sites_solution_primary .     Please Do !
```

   0 **ALERT level A** = Most likely a serious problem - resolve or explain
   0 **ALERT level B** = A potentially serious problem, consider carefully
   3 **ALERT level C** = Check. Ensure it is not caused by an omission or oversight
  12 **ALERT level G** = General information/check it is not something unexpected

   5 ALERT type 1 CIF construction/syntax error, inconsistent or missing data
   3 ALERT type 2 Indicator that the structure model may be wrong or deficient
   0 ALERT type 3 Indicator that the structure quality may be low
   5 ALERT type 4 Improvement, methodology, query or suggestion
   2 ALERT type 5 Informative message, check

It is advisable to attempt to resolve as many as possible of the alerts in all categories. Often the minor alerts point to easily fixed oversights, errors and omissions in your CIF or refinement strategy, so attention to these fine details can be worthwhile. In order to resolve some of the more serious problems it may be necessary to carry out additional measurements or structure refinements. However, the purpose of your study may justify the reported deviations and the more serious of these should normally be commented upon in the discussion or experimental section of a paper or in the "special_details" fields of the CIF. checkCIF was carefully designed to identify outliers and unusual parameters, but every test has its limitations and alerts that are not important in a particular case may appear. Conversely, the absence of alerts does not guarantee there are no aspects of the results needing attention. It is up to the individual to critically assess their own results and, if necessary, seek expert advice.

Publication of your CIF in IUCr journals

A basic structural check has been run on your CIF. These basic checks will be run on all CIFs submitted for publication in IUCr journals (*Acta Crystallographica*, *Journal of Applied Crystallography*, *Journal of Synchrotron Radiation*); however, if you intend to submit to *Acta Crystallographica Section C* or *E* or *IUCrData*, you should make sure that full publication checks are run on the final version of your CIF prior to submission.

Publication of your CIF in other journals

Please refer to the *Notes for Authors* of the relevant journal for any special instructions relating to CIF submission.

PLATON version of 10/08/2020; check.def file version of 06/08/2020

Datablock 1 - ellipsoid plot

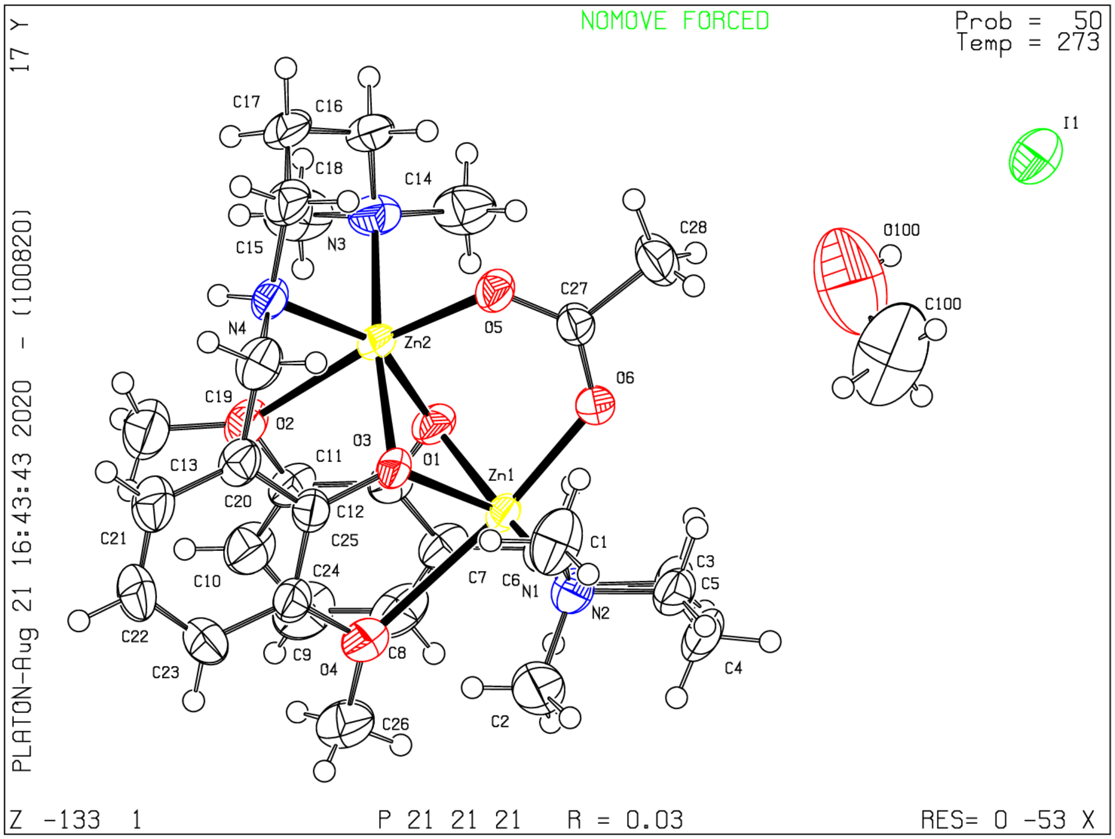

Datablock 2 - ellipsoid plot

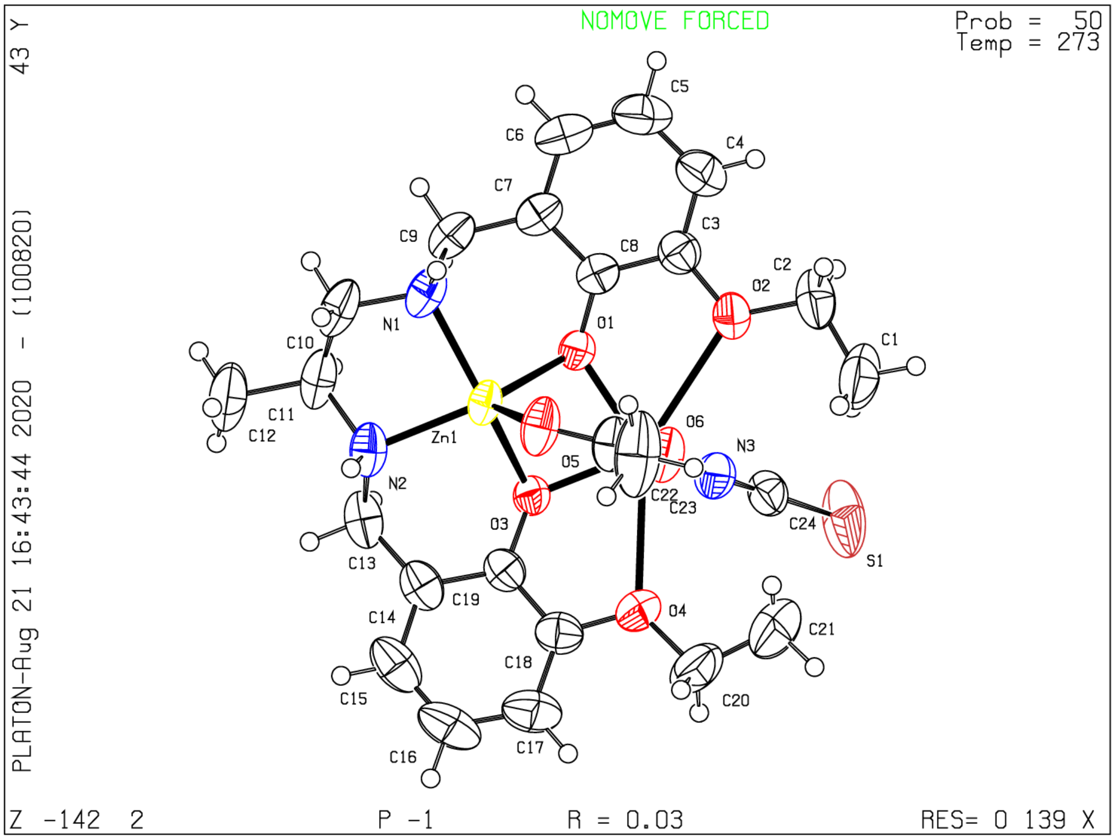

Supplement: Supplementary file 1 [file ijms-21-07091-s001.zip › checkcif.pdf]
