# Supplementary material for: Intramolecular Spodium Bonds in Zn(II) Complexes: Insights from Theory and Experiment
Source: Int J Mol Sci. 2020 Sep 25;21(19):7091. doi: 10.3390/ijms21197091 (PMC7582961; doi:10.3390/ijms21197091)
Supplement: Supplementary file 1 [file ijms-21-07091-s001.zip › Supplementary Information.pdf]

Electronic Supplementary Information

Intramolecular Spodium Bonds in Zn(II) Complexes: Insights from Theory and Experiment

Mainak Karmakar [1], Antonio Frontera [2], Shouvik Chattopadhyay [1,*], Tiddo J. Mooibroek [3,*], and Antonio Bauzá [2,*]

[1] Department of Chemistry, Inorganic Section, Jadavpur University, Kolkata 700 032, India; mainak31893@gmail.com

[2] Departament de Química, Universitat de les Illes Balears, Crta. de Valldemossa km 7.5, 07122 Palma (Baleares), Spain; toni.frontera@uib.es

[3] van 't Hoff Institute for Molecular Sciences, Universiteit van Amsterdam, Science Park 904, 1098 XH Amsterdam, The Netherlands

* Correspondence: shouvik.chem@gmail.com (S.C.); t.j.mooibroek@uva.nl (T.J.M.); antonio.bauza@uib.es (A.B.)

Table of contents

1. Additional structural description of compounds 1 and 2

2. Hirshfeld surface analyses

3. Spectral characterization

4. Complete QTAIM/NCIPlot of 1 and 2

5. References

1. Additional structural description of compounds 1 and 2

The X-ray crystal structure determination reveals that complex **1** crystallizes in the orthorhombic space group, *P*$2_12_12_1$, whereas complex **2** crystallizes in triclinic space group, *P*$\bar{1}$.. Important bond lengths and bond angles have been listed in Tables S1 and S2 respectively. In these two dinuclear complexes two zinc(II) centers are connected through a bridging($\mu_{1,3}$) acetate ligand.

[{Zn(L^1)}$_2$($\mu_{1,3}$-OAc)]I. CH$_3$OH (1)

A perspective view of complex **1** with the selective atom numbering scheme is shown in Figure S1 (see also Figure 1). Complex **1** is composed of a discrete dinuclear cation, [{Zn(L^1)}$_2$($\mu_{1,3}$-OAc)]$^+$ with one uncoordinated iodide counter anion. A lattice methanol molecule is also present. The catonic part of the complex consists of two units of reduced Schiff base ligands (deprotonated), one acetate {$\mu_{1,3}$ bridging} and two zinc(II) centers {Zn(1) and Zn(2)}. The zinc(II) centers are penta-coordinated with an N_2O_3 environment, as is also shown in Figure S2.

Both zinc ions are bonded to one tertiary amine nitrogen atom [N(1) for Zn(1) and N(3) for Zn(2)], one secondary nitrogen atom [N(2) for Zn(1) and N(4) for Zn(2)], two phenolate oxygen atoms [O(1) and O(2)] and one oxygen atom from bridging acetate ligand {O(6) for Zn(1) and O(5) for Zn(2)}. The geometry of any penta-coordinated metal centre may be measured using the trigonality index or Addison parameter, τ [τ = (β−α)/ 60 where β and α, are the two largest angles around the central atom; τ = 0 and 1 for perfect square pyramidal and trigonal bipyramidal geometries, respectively [1,2]. In the present complex, trigonality indices [τ = 0.34 for Zn(1) and τ = 0.33 for Zn(2)] suggest that the coordination geometry of each penta-coordinated zinc is a highly distorted square pyramidal. Thus, Zn(1) is equatorially coordinated by O(1), O(3), N(1) and N(2); and its axial position is occupied by O(6). Similarly, For Zn(2), the equatorial plane contains O(1), O(3), N(3) and N(4), whereas O(5) occupies the axial position. The displacements of the donor atoms from the Zn(1)O_2N_2 basal plane are 0.211(3), −0.266(3), 0.166(5) and −0.239(4) Å for O(1), O(3), N(1) and N(2) respectively. For Zn(2)O_2N_2 these values are 0.734(3), −0.888(3), −0.956(2) and 0.424(5) Å for O(1), O(3), N(3) and N(4) respectively. Both N-donor atoms connected to the Zn(II) ions are seperted by a propyl bridge, resulting in the two six-membered chelate rings as shown in Figure S3. Both rings have a chair conformation with puckering parameters [3] q = 0.688(5) Å, θ = 172.3(4)° and ϕ = 160(4)° in Zn(1)−N(1)−C(3)−C(4)−C(5)-N(2) and q = 0.650(6) Å, θ = 176.4(5)° ϕ = 128(7)° in Zn(2)−N(3)−C(16)−C(17)− C(18)-N(15).

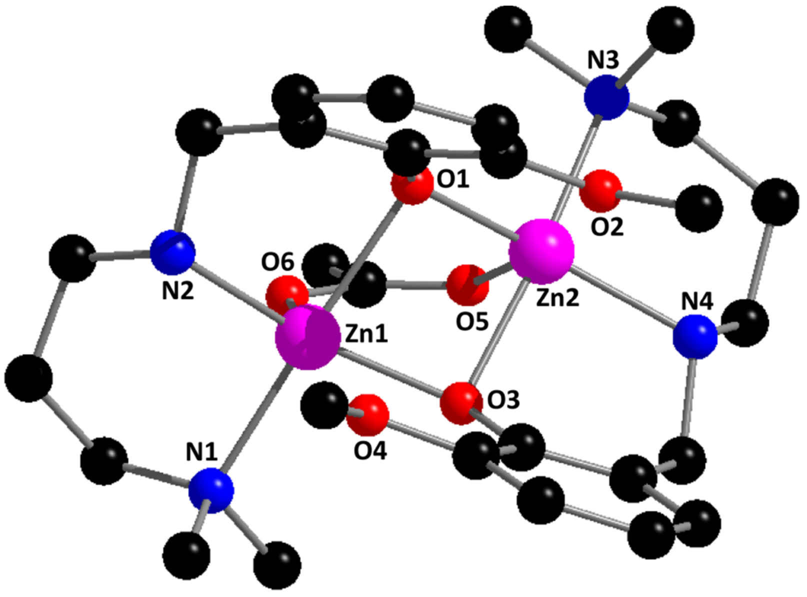

Figure S1. Perspective view of complex **1** with selective atom numbering schemes. Hydrogen atoms, anionic part (iodide) and lattice methanol molecule have been omitted for clarity.

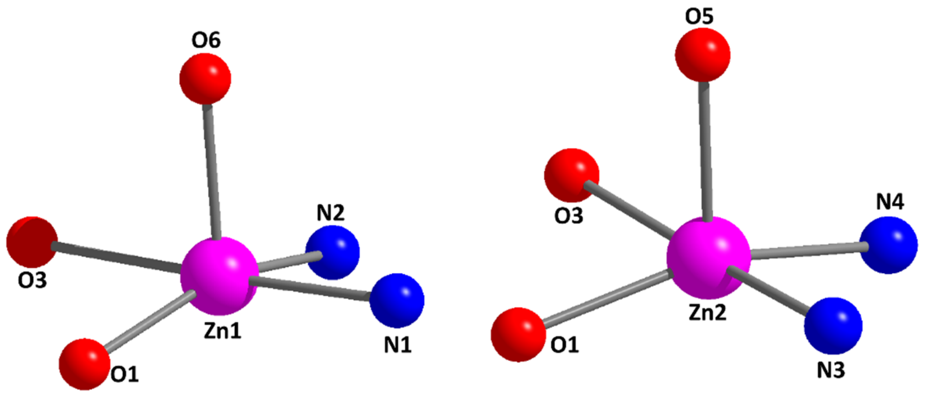

Figure S2. Coordination environments around Zn(1) and Zn(2) in complex **1**.

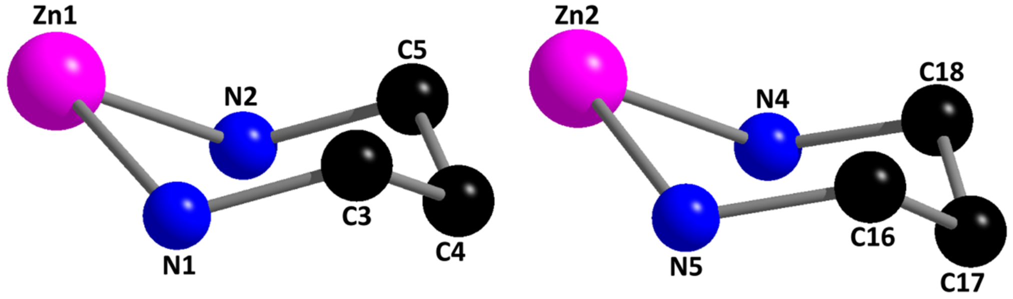

Figure S3. Saturated chelate rings in complex **1.**

[(L)Zn($\mu_{1,3}$-OAc)Zn(SCN)] (2)

A perspective view of complex **2** with the selective atom numbering scheme is shown in Figure S4 (see also Figure 1). Complex **2** is composed of one unit of deprotonated reduced Schiff base ligand (L^{2-}), one acetate [$\mu_{1,3}$ bridging], two zinc(II) centers [Zn(1) and Zn(2)] and one thiocyanate anion (SCN^{-}). The deprotonated reduced Schiff base ligand (L^{2-}) can be viewed as having two compartments: an 'inner' compartment with a N_2O_2 donor environment (N(1), N(2), O(1), O(3)) and an 'outer' compartment with four O-donor atoms (O(1)–O(4)). Zn(1) is situated at the inner compartment and additionally ligated by the bridging acetate (with O(5)). Zn(2) is located in the outer compartment, but only truly ligated by the bridging O(1) and O(3), and additionally by the bridging acetate (with O(6)) and the isothianate anion (with N(3)).

The trigonality index or Addison parameter (τ) of the penta-coordinated Zn(1) is 0.169, which means that it is a distorted square pyramidal. Zn(1) is equatorially coordinated by N(1), N(2), O(1), and O(3); and its axial position is occupied by O(5) [Figure S5a]. The displacements of the donor atoms from the Zn(1)O_2N_2 basal plane are 0.029(2), 0.200(2), 0.2045(17) and 0.0140(16) Å for N(1), N(2), O(1), and O(3) respectively.

Zn(2) is in a distorted tetrahedral geometry, being coordinated to two phenolate oxygen atoms of deprotonated reduced Schiff base [O(1) and O(3)], one oxygen of bridging acetate [O(6)] and the nitrogen atom [N(3)] of thiocyanate ligand (SCN^{-}) [Figure S5b]. The five membered chelate ring, Zn(1)–N(1)–C(10)–C(11)–N(2) assumes as envelop conformation [Figure S5c] with puckering parameters q = 0.451(3) Å and ϕ = 288.5(3)°.

The crystal packing of **2** is doninated by a hydrogen bond between the thiocyanate ligand S-atom and the secundary amine H-atom ofin N1, forming infinite chains along the crystallographic a-axis. No such strong intermolecular interactions were observed in the crystal packing of **1**. The solid state structures of both complexes were (further) stabilized by π···π and C–H···π interactions, as is detailed in Tables S3–S5.

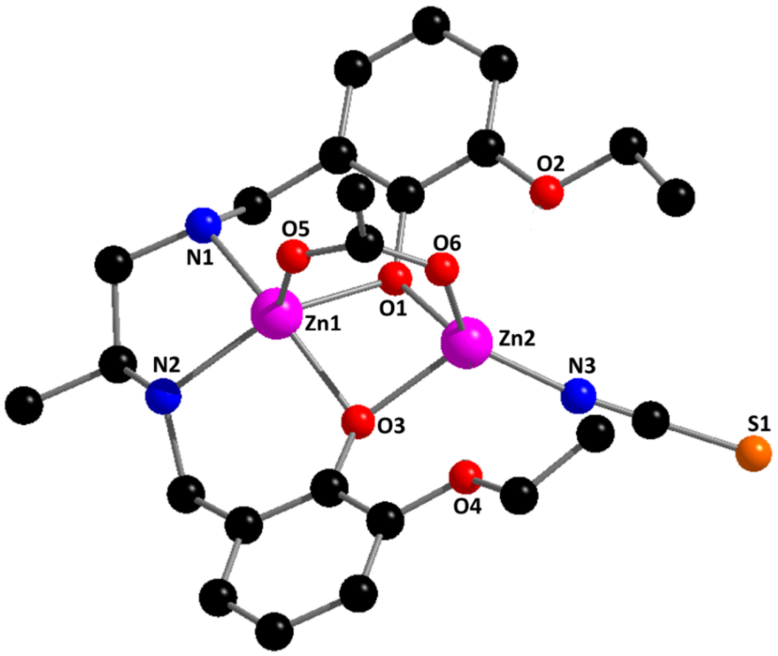

Figure S4. Perspective view of complex **2** with selective atom numbering schemes. Hydrogen atoms have been omitted for clarity.

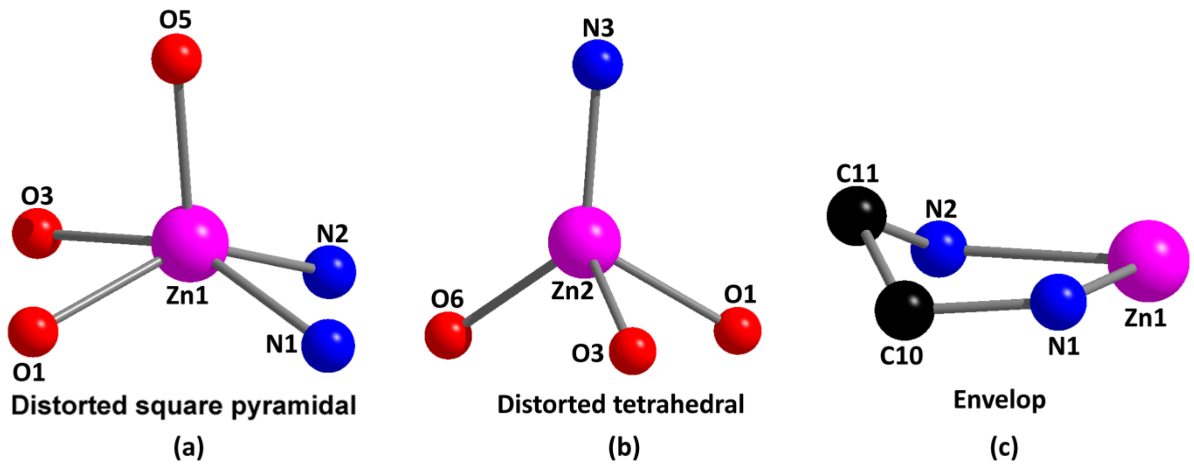

Figure S5. Coordination environments around (**a**) Zn(1) and (**b**) Zn(2) in complex **2**; (**c**) Saturated chelate rings in complex **2.**

Table S1. Selected bond lengths (Å) of complexes **1** and **2**.

Complex	1	2
Zn(1)-O(1)	2.212(4)	2.0581(18)
Zn(1)-O(3)	1.977(3)	2.0422(18)
Zn(1)-O(5)	-	1.978(2)
Zn(1)-O(6)	2.051(4)	-
Zn(1)-N(1)	2.169(5)	2.091(2)
Zn(1)-N(2)	2.075(4)	2.099(2)
Zn(2)-O(1)	1.983(4)	2.0167(18)
Zn(2)-O(3)	2.165(3)	2.0072(17)
Zn(2)-O(5)	2.075(4)	-
Zn(2)-O(6)	-	1.976(2)
Zn(2)-N(3)	2.160(5)	1.922(2)
Zn(2)-N(4)	2.086(4)	-

Table S2. Selected bond angles (°) of the complexes **1** and **2**.

Angle Name (Complex 1)	Angle Value	Angle Name (Complex 2)	Angle Value
O(1)-Zn(1)-O(3)	79.23(13)	O(1)-Zn(1)-O(3)	76.24(7)
O(1)-Zn(1)-O(6)	87.70(15)	O(1)-Zn(1)-O(5)	100.72(8)
O(1)-Zn(1)-N(1)	175.88(17)	O(1)-Zn(1)-N(1)	91.55(8)
O(1)-Zn(1)-N(2)	89.74(16)	O(1)-Zn(1)-N(2)	143.30(8)
O(3)-Zn(1)-O(6)	100.83(15)	O(3)-Zn(1)-O(5)	97.49(8)
O(3)-Zn(1)-N(1)	98.83(16)	O(3)-Zn(1)-N(1)	153.44(8)
O(3)-Zn(1)-N(2)	155.20(17)	O(3)-Zn(1)-N(2)	91.40(8)
O(6)-Zn(1)-N(1)	89.11(18)	O(5)-Zn(1)-N(1)	108.03(9)
O(6)-Zn(1)-N(2)	100.84(18)	O(5)-Zn(1)-N(2)	115.24(9)
N(1)-Zn(1)-N(2)	93.42(19)	N(1)-Zn(1)-N(2)	84.46(9)
O(1)-Zn(2)-O(3)	80.27(12)	O(1)-Zn(2)-O(3)	77.96(7)
O(1)-Zn(2)-O(5)	100.88(15)	O(1)-Zn(2)-O(6)	101.78(8)
O(1)-Zn(2)-N(3)	97.36(17)	O(1)-Zn(2)-N(3)	117.19(9)
O(1)-Zn(2)-N(4)	154.34(17)	O(3)-Zn(2)-O(6)	104.25(8)
O(2)-Zn(2)-N(3)	92.86(15)	O(3)-Zn(2)-N(3)	115.48(9)
O(3)-Zn(2)-O(5)	87.59(13)	O(6)-Zn(2)-N(3)	128.37(9)
O(3)-Zn(2)-N(3)	174.27(16)	-	-
O(3)-Zn(2)-N(4)	90.00(15)	-	-
O(5)-Zn(2)-N(4)	102.38(17)	-	-
N(3)-Zn(2)-N(4)	94.25(19)	-	-

Table S3. Hydrogen bond distance (Å) and angles (°) of complex **1** and **2**.

Complex	D-H···A	D-H	H···A	D···A	∠D-H···A
1	O(100)–H(100)···I(1)	0.82	2.72	3.529(3)	168
2	N(1)–H(2N)···N(5) [a]	0.89(4)	2.51(4)	3.382(3)	169(3)

D, donor; H, hydrogen; A, acceptor. Symmetry transformation [a] = 1+X, Y, Z.

Table S4. Geometric features (distances in Å and angles in °) of the C-H···π interactions obtained for complexes **1–3**.

Complex	C-H···Cg(Ring)	H···Cg (Å)	C-H···Cg (°)	C···Cg (Å)
1	C(1)-H(1A)···Cg(2)	2.99	113	3.474(7)
	C(2)-H(2A)···Cg(2)	2.80	115	3.319(8)
	C(15)-H(15A)···Cg(3)	2.71	116	3.265(9)
2	C(13)-H(13B)···Cg(9)[b]	2.55	162	3.485(3)

Symmetry transformations: [b] = 1-X, 1-Y, 1-Z

For complex **1**:

Cg(2) = Centre of gravity of the ring [Zn(1)-O(3)-C(25)-C(24)-0(4)] and Cg(3) = Centre of gravity of the ring [Zn(2)-O(1)-C(12)-C(11)-O(2)].

For complex **2**:

Cg(9) = Centre of gravity of the ring [C(3)-C(4)-C(5)-C(6)-C(7)-C(8)].

Table S5. Geometric features (distances in Å and angles in °) of the π···π stacking in complexes **1.**

Complex	Cg(I)···Cg(J)	distance (Å)	α	Cg(I)···Prep	Cg(J)···Prep	Slippage
1	Cg(2)···Cg(3)	2.780(3)	31.58(18)	2.6873(17)	−2.6587(17)	-
	Cg(2)···Cg(10)	3.810(4)	28.2(3)	2.8225(17)	−3.304(3)	-
	Cg(3)···Cg(2)	2.780(3)	31.58(18)	−2.6587(17)	2.6873(17)	-
	Cg(3)···Cg(11)	3.898(3)	31.1(2)	−2.7084(17)	3.369(2)	-
	Cg(10)···Cg(2)	3.810(4)	28.2(3)	−3.304(3)	2.8225(17)	-
	Cg(11)···Cg(3)	3.898(3)	31.1(2)	3.368(2)	−2.7084(17)	-

Cg(2) = Centre of gravity of the ring [Zn(1)-O(3)-C(25)-C(24)-0(4)]; Cg(3) = Centre of gravity of the ring [Zn(2)-O(1)-C(12)-C(11)-O(2)]; Cg(10) = Centre of gravity of the ring [C(7)-C(8)-C(9)-C(10)-C(11)-C(12)] and Cg(11) = Centre of gravity of the ring [C(20)-C(21)-C(22)-C(23)-C(24)-C(25)].

2. Hirshfeld surfaces analysis

Hirshfeld surfaces [4,5] and the associated 2D-fingerprint [6,7] plots were calculated using Crystal Explorer [8] from the respective CIF files. Bond lengths to hydrogen atoms were set to standard values. For each point on the Hirshfeld isosurface, two distances were defined: d_e, the distance from the point to the nearest nucleus external to the surface; and d_i, the distance to the nearest nucleus internal to the surface. The normalized contact distance (d_{norm}) based on d_e and d_i is given by Equation S1:

$$d_{norm} = \frac{(d_i - r_i^{vdw})}{r_i^{vdw}} + \frac{(d_e - r_e^{vdw})}{r_e^{vdw}} \quad \text{(S1)}$$

where r_i^{vdw} and r_e^{vdw} were the van der Waals radii of the atoms involved. The value of d_{norm} is negative for an intermolecular contact (shorter than the van der Waals separations). The parameter d_{norm} is displayed as a surface with a red-white-blue colour scheme, where bright red spots highlight a negative d_{norm} (i.e., a shorter contact). For a given crystal structure and set of spherical atomic electron densities, the Hirshfeld surface was unique[4] and it was this property that suggested the possibility of gaining additional insight into the intermolecular interaction of molecular crystals.

Hirshfeld surfaces of the complex mapped over d_{norm}, shape index and curveness (range of −0.1 to 1.5 Å) are illustrated in Figure S6. Red spots on these surfaces denote the dominant interactions [N···H/H···N, C···H/H···C, O···H/H···O, S···H/H···S and I···H/H···I].

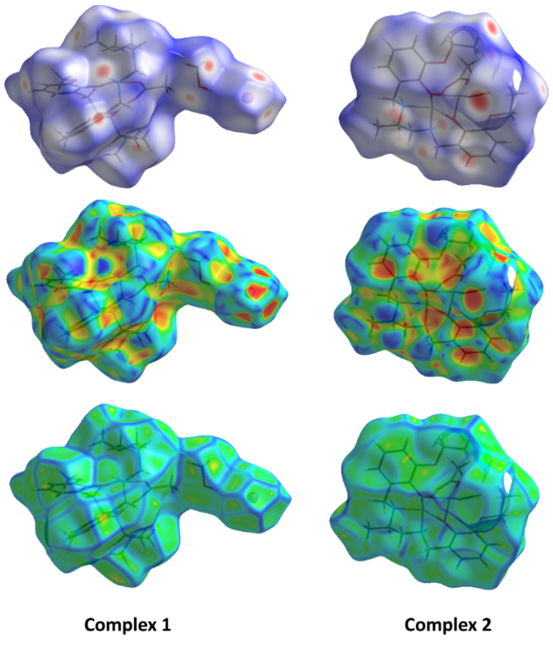

Figure S6. Hirshfeld surfaces of complexes **1** and **2** mapped over d_{norm} (top), shape index(middle) and curveness (bottom).

The 2D fingerprint plots [9] revealed that, from a numerical point of view, the main intermolecular interactions in complex **1** are O···H/H···O, C···H/H···C and I···H/H···I. For complex **2** the main intermolecular interactions are C···H/H···C, O···H/H···O, N···H/H···N and S···H/H···S in Figure S7.

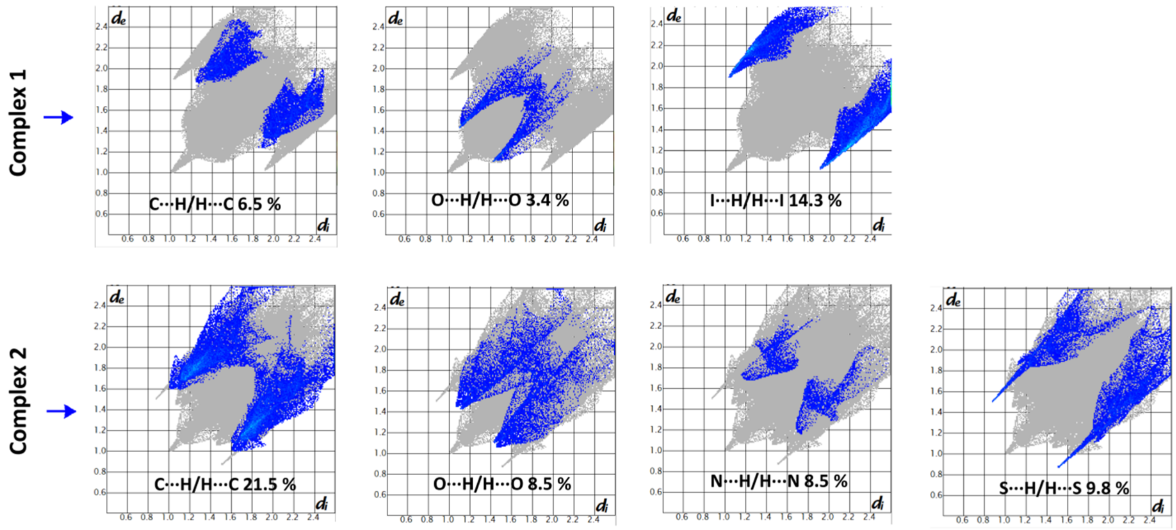

Figure S7. Fingerprint plot: Different contacts contributed to the total Hirshfeld Surface area of the complexes **1** and **2.**

3. Spectral Characterization

The IR-spectral data of each of the complexes are consistent with their respective crystal structure (see Figure S8). Absence of any band characteristic to azomethine (>C=N) stretching vibrations [10] around 1600 cm^{-1} in the IR spectra of the complexes **1** and **2** confirms the formation of the reduced Schiff base ligands. Further evidence in favor of presence of reduced Schiff base ligands in the complexes is supported by the prescence of characteristic N–H stretching broad bands around 3150–3250 cm^{-1} [11–14]. Characteristic bands in the range of 2800–3000 cm^{-1} for C–H stretching are present in the IR spectra of both complexes [15]. Strong and sharp bands characteristic to symmetric and antisymmetric stretching of the carboxylate groups re observe at 1473 cm^{-1} and 1557 cm^{-1} (for complex **1**) and 1472 cm^{-1} and 1566 cm^{-1} (for complex 2) [16–19]. Existence of small and broad band in the IR spectra of complex **1** around 3390 cm^{-1} characteristic to O–H stretching, confirms the presence of lattice methanol moleule [20]. A strong and sharp band around 2102 cm^{-1} in the IR spectrum of complex **2** indicates the presence of metal bound (N-bonded) thiocyanate [21–24].

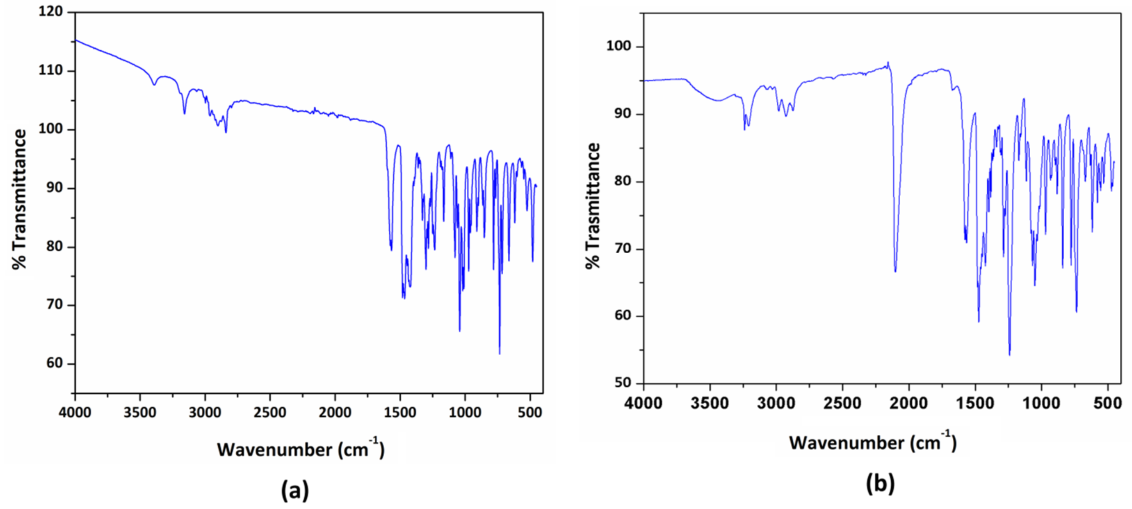

Figure S8. IR spectra of complexes (**a**) **1** and (**b**) **2**.

The electronic spectra of both complexes in DMF medium are very similar to each other (see Figure S9). Complex **1** shows broad a band around 291 nm and complex **2** shows broad a band around 286 nm corresponding to intra ligand charge transfer transition.

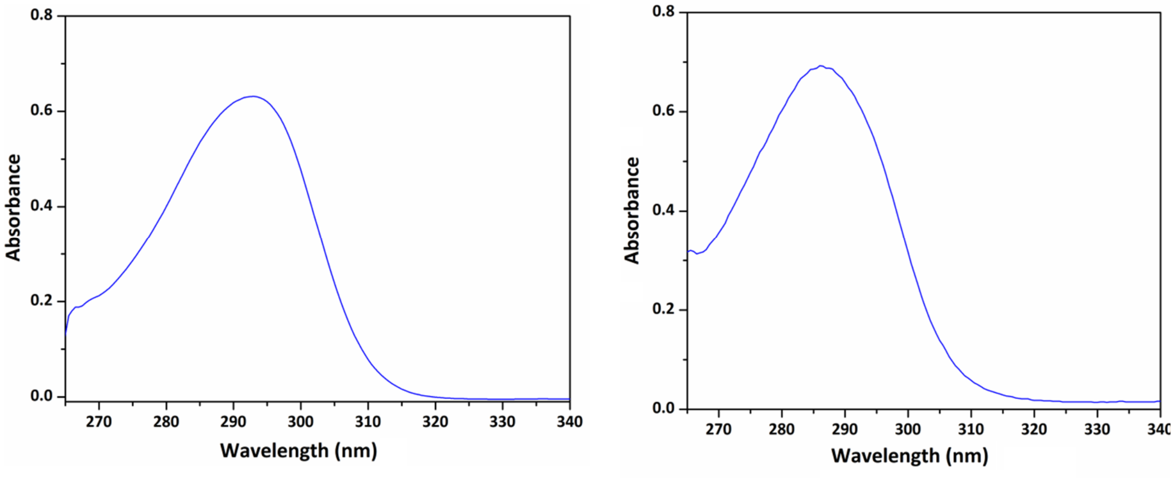

(**a**) (**b**)

Figure S9. Electronic absorption spectra of complexes (**a**) **1** and (**b**) **2**. No bands over 340 nm are observed.

The X-ray powder diffraction patterns of both compounds are given in Figure S10 (red), showing a good agreement with the simulated spectra (green).

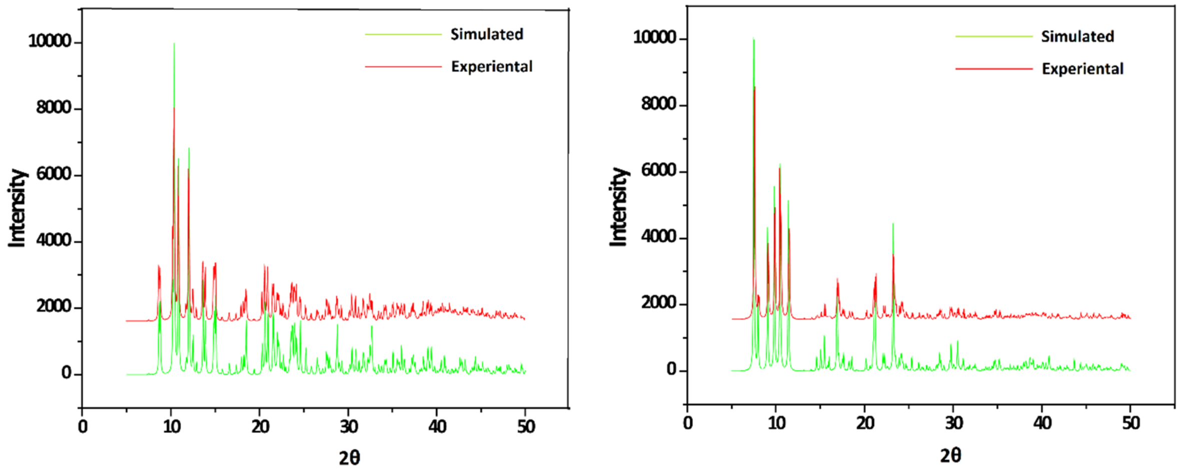

(a) **(b)**

Figure S10. PXRD patterns of (**a**) complex **1** and (**b**) complex **2.**

4. Complete QTAIM/NCIPlot of 1 and 2

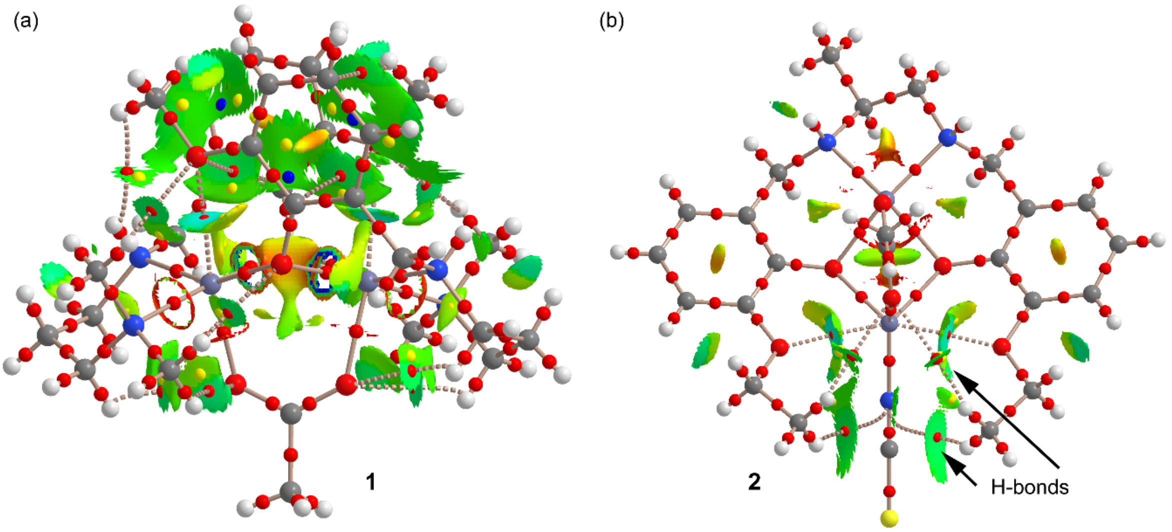

Figure S11. Complete QTAIM/NCIplot analyses of complexes 1 (**a**) and 2 (**b**).

5. References

1. Stewart, J.J.P. Optimization of parameters for semiempirical methods I. Method *J. Comput. Chem*. **1989**, *10*, 209–220.
2. Addison, A.W.; Rao, T.N.; Reedjik, J.; van Rinj, J.; Verschoor, G.C. Synthesis, structure, and spectroscopic properties of copper(II) compounds containing nitrogen–sulphur donor ligands; the crystal and molecular structure of aqua[1,7-bis(N-methylbenzimidazol-2′-yl)-2,6-dithiaheptane]copper(II) perchlorate *J. Chem. Soc., Dalton Trans*. **1984**, *7*, 1349–1356.
3. Cremer, D.; Pople, J.A. General definition of ring puckering coordinates *J. Am. Chem. Soc*. **1975**, *97*, 1354–1358.
4. McKinnon, J.J.; Spackman, M.A.; Mitchell, A.S. Novel tools for visualizing and exploring intermol-ecular interactions in molecular crystals. *Acta Crystallogr*. **2004**, *B60*, 627–668.
5. McKinnon, J.J.; Jayatilaka, D.; Spackman, M.A. Towards quantitative analysis of intermolecular interactions with Hirshfeld surfaces. *Chem. Commun*. **2007**, *37*, 3814–3816.
6. Spackman, M.A.; Jayatilaka, D. Hirshfeld surface analysis. *CrystEngComm* **2009**, *11*, 19–32.
7. Spackman, M.A. Molecular electric moments from x-ray diffraction data. *Chem. Rev*. **1992**, *92*, 1769–1797.
8. Turner, M.J.; McKinnon, J.J.; Wolff, S.K.; Grimwood, D.J.; Spackman, P.R.; Jayatilaka, D.; Spackman, M.A. CrystalExplorer17. University of Western Australia: Perth, 2017, Available online: http://hirshfeldsurface.net.
9. Spackman, M.A.; McKinnon, J.J. Fingerprinting intermolecular interactions in molecular crystals. *CrystEngComm* **2002**, *4*, 378–392.
10. Elemike, E.E.; Nwankwo, H.U.; Onwudiwe, D.C.; Hosten, E.C. Synthesis, crystal structures, quantum chemical studies and corrosion inhibition potentials of 4-(((4-ethylphenyl)imino)methyl)phenol and (E)-4-((naphthalen-2-ylimino) methyl) phenol Schiff bases. *J. Mol. Struct*. **2017**, *1147*, 252–265.
11. Basak, T.; Ghosh, K.; Chattopadhyay, S. Synthesis, characterization and catechol oxidase mimicking activity of two iron(III) schiff base complexes. *Polyhedron* **2018**, *146*, 81–92.
12. Chattopadhyay, S.; Ray, M.S.; Chaudhuri, S.; Mukhopadhyay, G.; Bocelli, G.; Cantoni, A.; Ghosh, A. Nickel(II) and copper(II) complexes of tetradentate unsymmetrical Schiff base ligands: First evidence of positional isomerism in such system*Inorg. Chim. Acta* **2006**, *359*, 1367–1375.
13. Socrates, G. *Infrared and Raman Characteristic Group Frequencies. Tables and Charts*; John Wiley & Sons: Chichester, UK, 2001; pp.108–113.
14. Karmakar, M.; Chattopadhyay, S. Synthesis, structure and nitroaromatic sensing ability of a trinuclear zinc complex with a reduced Schiff base ligand: Assessment of the ability of the ligand to sense zinc ion. *Polyhedron*, **2020**, *187*, 114639.

15. Roy, S.; Basak, T.; Khan, S.; Drew, M.G.B.; Bauza, A.; Frontera, A.; Chattopadhyay, S. A Combined Experimental and Theoretical Study on the Formation of a Cyclic Tetrameric Water Cluster and a Similar Type of Cyclic Cluster in Copper(II) Schiff Base Complexes. *ChemistrySelect* **2017**, *2*, 9336–9343.
16. Ghosh, K.; Harms, K.; Bauzá, A.; Frontera, A.; Chattopadhyay, S. Heteronuclear cobalt(iii)/sodium complexes with salen type compartmental Schiff base ligands: methylene spacer regulated variation in nuclearity. *Dalton Trans.* **2018**, *47*, 331–347.
17. Sharma, A.K.; Lloret, F.; Mukherjee, R. Phenolate- and Acetate (Both μ2-1,1 and μ2-1,3 Modes)-Bridged Linear Co^{II}_3 and $Co^{II}_2Mn^{II}$ Trimers: Magnetostructural Studies. *Inorg. Chem.* **2013**, *52*, 4825–4833.
18. Cristóvão, B.; Miroslaw, B.; Kłak, J.; Rams, M. Carbonato-bridged heteronuclear $Ni^{II}_2Ln^{III}_2$ (Ln = Tb, Dy, Ho, Er, Tm, Yb, Lu) complexes synthesized by fixation of atmospheric CO2–Structural and magnetic studies. *Polyhedron* **2015**, *85*, 697–704.
19. Karmakar, M.; Chattopadhyay, S. *Polyhedron*, **2020**, *184*, 114527–114537.
20. Bhattacharyya, A.; Harms, K.; Chattopadhyay, S. Formation of T4(1) water tapes interconnected via centrosymmetric nickel(II) Schiff base complex to produce a 3D architecture. *Inorg. Chem. Commun*. **2014**, *48*, 12–17.
21. Jana, S.; Bhowmik, P.; Das, M.; Jana, P.P.; Harms, K.; Chattopadhyay, S. Synthesis and characterisation of two double EE azido and thiocyanato bridged dimeric Cu(II) complexes with tridentate Schiff bases as blocking ligands. *Polyhedron* **2012**, *37*, 21–26.
22. Bhowmik, P.; Nayek, H.P.; Corbella, M.; Alcalde, N.A.; Chattopadhyay, S. Control of molecular architecture by steric factors: mononuclear vs polynuclear manganese(iii) compounds with tetradentate N2O2 donor Schiff bases. *Dalton Trans*. **2011**, *40*, 7916–7926.
23. Bhattacharyya, A.; Roy, S.; Chakraborty, J.; Chattopadhyay, S. Two new hetero-dinuclear nickel(II)/zinc(II) complexes with compartmental Schiff bases: Synthesis, characterization and self assembly. *Polyhedron* **2016**, *112*, 109–117.
24. Karmakar, M.; Roy, S.; Chattopadhyay, S. A series of trinuclear zinc(ii) complexes with reduced Schiff base ligands: turn-off fluorescent chemosensors with high selectivity for nitroaromatics. *New. J. Chem*. **2019**, *43*, 10093–10102.
